# Sustainability practices of accommodation sector representatives: The case of mountain protected area Serra da Estrela Natural Park (Portugal)

**DOI:** 10.1371/journal.pone.0347472

**Published:** 2026-04-20

**Authors:** Ilinca-Valentina Stoica, Luis Santos, Alexandra Cioclu, Ioan-Adrian Toma, Daniela Zamfir

**Affiliations:** 1 Interdisciplinary Center for Advanced Research on Territorial Dynamics (CICADIT), University of Bucharest, Bucharest, Romania; 2 Faculty of Geography, University of Bucharest, Bucharest, Romania; 3 Department of Archaeology, Conservation, Restoration and Heritage, Polytechnic Institute of Tomar, Tomar, Portugal; 4 Geosciences Research Centre, Coimbra University, Coimbra, Portugal; 5 Simion Mehedinti Doctoral School, University of Bucharest, Bucharest, Romania; University of Naples Federico II: Universita degli Studi di Napoli Federico II, ITALY

## Abstract

Research on the commitment of accommodation representatives to sustainability practices in protected areas is limited. In recent years, digitisation has become increasingly important in promoting places and the decision-making process for travel. However, there is still a lack of knowledge about the accuracy of the sustainability practices claimed by accommodation establishments on online booking platforms. Focusing on Serra da Estrela Natural Park in Portugal as a case study, the current approach is the first one to verify, through interviews with the accommodation representatives, the sustainability practices declared on Booking.com. For deeper insights, the perception of accommodation establishment representatives regarding the role of sustainability in their business is explored. To obtain a comprehensive perspective, a sustainability practice ranking is employed, with the established hierarchy spatially represented through GIS techniques. Even though the results confirmed the largest majority of the reported information on Booking.com, the information is insufficient, as additional practices emerged through interviews. Challenging the results of other studies, the commitment to sustainability is not correlated either with the size of the unit or its degree of comfort, denoting rather a correlation with personal values, beliefs and business strategy. Overall, most respondents express a broader, multi-scalar perspective on sustainability, shaped by their awareness of the businesses’ embeddedness in the natural park, which features outstanding natural values that need preservation. Furthermore, several categories of opinions on the benefits and downsides of developing a business within a protected area are highlighted. A notable finding is that half of the respondents considered the regulations imposed by the protected area status as essential to prevent chaotic development and environmental degradation. However, the interaction with natural park representatives is described as mostly passive, since most respondents have little to no interaction with them, highlighting the need to establish mutually beneficial cooperation and foster pro-sustainability behaviour.

## Introduction

Protected areas are crucial for conserving natural resources, preventing biodiversity loss and promoting human well-being [1]. Nevertheless, Jones et al. [2] discovered that 32.8% of protected area land globally are subject to intense anthropic pressure (among which tourism), with many located in Western Europe. In this regard, there is a growing demand for recreational activities and tourism in protected areas [3], also boosted by the Covid-19 pandemic effects.

Many protected areas overlap with rural remote areas affected by various problems, such as communities in need of alternative livelihoods in the context of declining traditional economic activities, the weakening of the economic system amid depopulation and demographic ageing, loss of cultural heritage, etc. In this context, tourism can lead to the creation of jobs, income for the local population, and boost new business development and revenues for entrepreneurs, including in related sectors [4–6]. As a result, tourism contributes to the overall development of the local economy. Additionally, adequately managed tourism activities can increase environmental awareness and serve as a tool for enhancing biodiversity conservation [1,7].

Nevertheless, previous studies have shown that poorly managed tourism can lead to environmental degradation due to high resource consumption (e.g., water, energy), waste production, greenhouse gas emissions, pollution [8–12], biodiversity loss [1] and land-use changes [3]. As a result of concerns about the lasting negative impact of this industry, resource efficiency and environment preservation are advocated through a proactive approach and the adoption of sustainability practices by businesses [10,13,14]. Moreover, in protected areas, a delicate balance must be found between tourism-related activities and the sustainable use of natural resources, which must be carefully managed to support the conservation goals set through the management plans [3]. Owners/managers of tourist accommodation establishments are among the key actors operating in protected areas that can contribute to achieving sustainable tourism. In this regard, they can influence the way tourism is practiced locally, and are perceived as potential agents of change that can transform tourists’ behaviour and attitudes towards resource preservation [15].

Still, there is limited knowledge about the commitment of accommodation representatives to sustainability practices in protected areas. Font et al. [16] analyse, from a cross-cutting international perspective, based on a self-reported survey, the practices and drivers of sustainability of tourism SMEs in 57 European protected areas, which reveals heterogeneous results. At the regional level, Silik et al. [17] explore the environmental performance of accommodation units in 16 protected areas in the Bolu province of Turkey through questionnaires. The authors advocate the need to increase the performance of establishments by applying several environmental practices. Similar conclusions were reached by Erdogan & Tosun (2009) [5], who investigated accommodation unit behaviour in the Goreme Historical National Park (Turkey). In addition, Armas-Cruz et al. [18] focus on the purpose, motivations and barriers to the implementation of environmental measures in the Fuerteventura Biosphere Reserve (Canary Islands, Spain), finding a medium-high commitment of accommodation establishments. Each of these studies investigated several sustainability measures and concluded that although valuable, the results could not be generalised, emphasising the need for more studies in other protected areas.

Still, collecting data on a large scale can be expensive and time-consuming. Against this background, there is a need to assess the sustainability practices of the accommodation sector through broadly comparable data in an accurate and cost-effective way [13]. Lately, online platforms are an increasingly popular tool for booking accommodation worldwide [19,20], and in this respect, they can play a key role in raising awareness among tourists and entrepreneurs for more sustainable tourism [20]. Previous findings have highlighted that communicating sustainability practices within large web portals could have a deeper impact, creating a positive portrayal of the business and competitive advantage [11,21,22] and thus directly influencing competitors to adopt similar measures [13]. This is also driven by increasing awareness of environmental issues and, in this context, the changing expectations of tourists for the implementation of green practices [10,17,19,21,23].

So far, few studies have examined how sustainability issues are communicated through online platforms by accommodation representatives or how this information can be used to analyse sustainable tourism in specific areas. In this regard, an earlier approach explored the integration degree of sustainability issues by testing eight online booking platforms and analysing the existing filters by selecting three types of tourism practised in nine destinations in the world [20]. In a rather similar study of accommodation establishments in Romania, Foris et al. [19] analyse four booking platforms, two of which are international (Booking.com and TripAdvisor), through the lens of environmental practices filters. As a common denominator, these studies concentrate more on how sustainability information is displayed.

From a different perspective, Rutecka et al. [6] stated that they had conducted the first research examining tourist establishments in an area according to declared sustainability practices on Booking.com, focusing on Poland. In a broader context, Borges-Tiago et al. [24] analyse the communication of sustainability initiatives, including eco-labels, in a region of Portugal by corroborating several online sources, among which booking engines (such as Booking.com, TripAdvisor, and Airbnb) from a multi-stakeholder perspective. Another trend is to directly investigate hotel websites to identify what green practices they implement [12,14,25], but some limitations were found related to the heterogeneity of information and the impossibility of making reliable comparisons.

Overall, one of the identified challenges is that not enough information about sustainable development is integrated on online platforms [20]. Additionally, it is unclear whether all measures adopted at the accommodation unit level are communicated [7,12] or whether all stated practices are actually implemented [25]. Thus, previous studies have pointed out that the topic of accommodation establishments’ declared environmental practices on online booking platforms is under-researched. In this regard, there is a pressing need to gain a better understanding of how the information is displayed, its feasibility and how it can be used to analyse certain areas [6,13,19].

The present research addresses this gap beyond previous approaches in that, to the best of our knowledge, this is the first study to verify, through interviews, the sustainability practices declared by accommodation establishment’s representatives on online booking platforms. Secondly, it investigates in depth how respondents perceive sustainability and its role in their business, in relation to the context of a NP (natural park) and the relationship with its representatives. Thus, given the recognised scarcity of knowledge, this research aimed to provide a better understanding of the perspective of accommodation establishments operating within a sensitive, protected area. Additionally, a sustainability practice ranking is used to gain a comprehensive perspective.

Overall, our contribution aims to provide insights into the exploration of information reported through digital platforms and their relevance for analysing sustainable tourism in different areas. Moreover, the results should provide knowledge valuable in strategic planning for tourism development in protected areas.

## Methodology

### Study area

**Fig 1:**
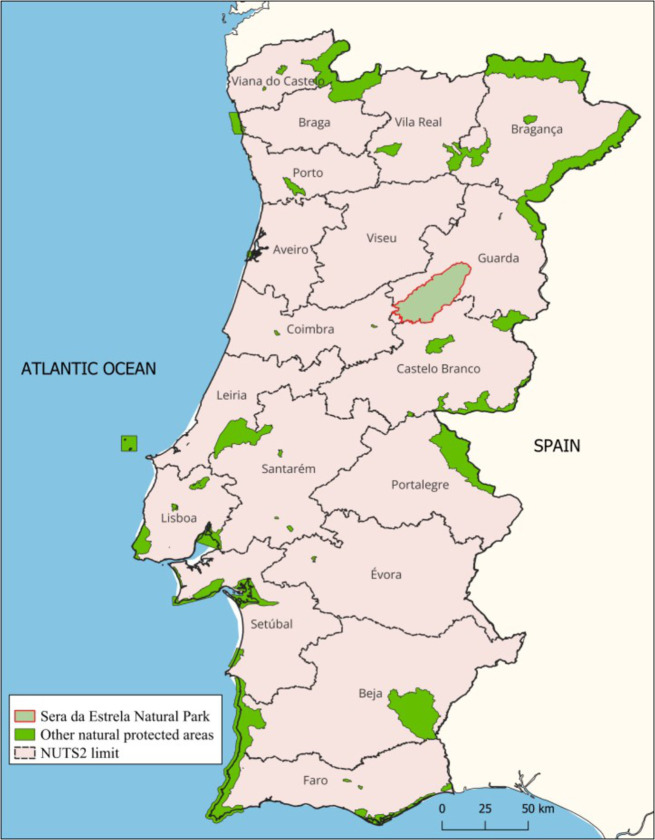
Geographical location of Serra da Estrela Natural Park. Source: Original map using European Protected Areas from European Environment Agency (https://www.eea.europa.eu/en/analysis/maps-and-charts/european-protected-areas-1) under CC-BY license and Natural Earth administrative boundaries (https://www.naturalearthdata.com/).

Serra da Estrela is a mountainous massif located in central-eastern Portugal (Fig 1), that includes the highest point of continental Portugal, Torre (1,993 m), and is part of the Iberian Central Cordillera. The significant importance of this territory was recognised in 1976 when it was granted the status of Natural Park. Since 2007 when its boundaries were reorganised, it covers 891.64 km^2^ [26].

From an administrative point of view, the Serra da Estrela Natural Park (SENP) territory overlaps over 56 freguesias (Parishes – smallest territorial units) comprised in six municipalities (Celorico da Beira, Covilhã, Gouveia, Guarda, Manteigas and Seia) in two districts (Castelo Branco and Guarda). Regarding the population, approximately 25,000 people live in the SENP area, according to the 2021 census [26]. Almost the entire territory is part of the Natura 2000 network as a site of community importance (SCI), while the upper plateau and Zêzere River upper course are declared wetlands of international importance under the Ramsar Convention [27]. In addition, in 2020, the UNESCO Serra da Estrela Geopark was established, covering a much larger area of about 2,216 km^2^, partially overlapping the territory of the SENP [28].

The SENP is characterised by diverse natural resources whose synergistic action has shaped a unique and enchanting landscape. Scattered throughout this vast natural area are picturesque villages, some of which have historical and cultural significance, creating an eco-cultural mosaic of ecological and cultural importance [29].

Although this region is historically associated with wool and cheese production, the tourism sector has grown in recent decades (especially in the last 10 years), being seen as a means of promoting economic and social development [30]. In Manteigas, the only municipality located entirely within the SENP, has seen an increase of 73% in the number of guests registered in tourist accommodation units between 2016 and 2022 [31]. Traditionally, tourism has been seasonal and geographically concentrated, with greater demand on weekends and during winter when snow falls [28]. In this regard, a representative feature is that it is the only place in Portugal with some snow tourism and the only ski resort in the country [32]. All these specific characteristics have increased the area’s renown and attractiveness.

### Data and methods

**Fig 2:**
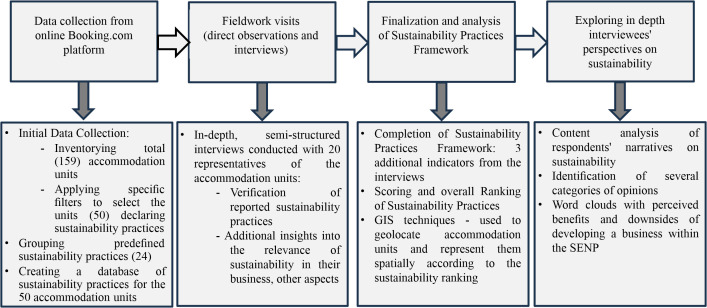
Analysis workflow.

The methodology consisted of several phases, as outlined in Fig 2. In the first phase, the initial dataset of tourist accommodation offers declaring sustainability practices in SENP was collected from Booking.com. The choice of this online platform is rooted in a previous study developed in the frame of H2020-MSCA-RISE HIGHLANDS.3 Project [33]. That research analysed several booking websites and found that Booking.com provides the most extensive information on the sustainability measures reported by accommodation units. In the same vein, Foris et al. [19], comparing four booking platforms, including Booking.com and TripAdvisor, find that Booking.com offers tourists more environmental filters.

Thus, from the website of this online travel agency, at the beginning, all the existing accommodation offers in SENP were sorted, resulting in 159 units. Then, the initial database was refined using specific filters to select accommodation units that declared they followed sustainability practices, resulting in a sample of 50 establishments.

**Fig 3:**
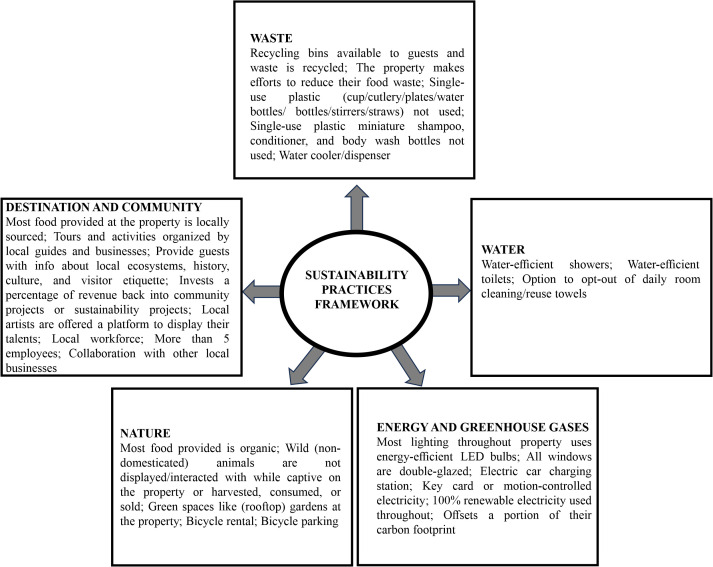
Sustainability practices framework (categories and indicators/practices) as resulted from Booking.com and interviews, used for analysis. Source: Booking.com and face to face interviews.

The 30 practices displayed in the Booking.com platform were grouped into 24 indicators (Fig 3), as six measures related to single-use plastic were grouped into only one, and two other measures (option to reuse towels/to give up daily room cleaning) were merged into one, given that they share the same features.

Subsequently, by simulating a booking, the characteristics of each accommodation were analysed in the framework of the declared sustainability practices and a database was created. The data were publicly displayed on Booking.com and manually extracted by two researchers for each unit in early January 2023. The sustainability practices listed on the platform were organised into five predefined categories: waste, water, energy and greenhouse gases, destination and community, and nature. The collection and analysis method complied with the terms and conditions for the source of the data.

In the second phase of the study, fieldwork visits were carried out for direct observations and in-depth, semi-structured interviews were conducted with the representatives of selected accommodation units (owners/managers) between January 15, 2023 and February 16, 2023. The data was used to verify the sustainability practices reported by accommodation establishments via Booking.com. At the same time, additional information was collected to understand the relevance of sustainability in their business, the main features of the unit, the implications of running their activity in a NP for furthering sustainable tourism, assessment of the interactions and collaborations with the NP’s representatives, motivation of tourists to choose this area.

Before starting the research, approval was obtained from the Ethics Committee. However, it is important to note that ethical considerations were outlined in the Grant Agreement of the HIGHLANDS.3 Project from the outset. As a result, the study adhered to both the grant agreement provisions and the ethics committee notice during data collection, following appropriate ethical protocols. After providing detailed information about the research, all participants signed an informed consent form before answering questions from the interview guide. The data collected was maintained in strict confidentiality and used only for academic research, ensuring that no personal information was disclosed to any third parties.

The interviews were conducted face to face over several weeks at the accommodation establishments by two of the researchers. The duration of the interviews ranged from 60 to 100 minutes, and they were audio recorded. Afterwards, they were transcribed verbatim and utilised for content analysis. Even though consent was obtained from the interviewees to use the information, the authors decided to maintain their anonymity by using random coding. The interviewees were assured that their responses would remain confidential and that they had the right to end the interview and their participation in the research at any time without consequences. Additionally, they were informed that responses would be analysed in an aggregate and that anonymised direct quotations might be included in publications.

The interviews were applied to representatives of 20 units declaring sustainability practices on Booking.com, as some of the accommodations were closed due to the seasonality of the tourist offer. In several situations, the owners/managers were not on site or could not be contacted at the time the research was carried out. In any case, it can be considered that data saturation has been reached, as the interview has stopped providing new relevant information.

In the third phase of the research, the sustainability practices framework was finalised from a comprehensive perspective to enable an overall analysis. Thus, the 24 indicators from the Booking.com platform were complemented with three representative indicators resulting from the data collected during the interviews for a better understanding of the economic and social impact. These three indicators relate to the involvement of the local workforce, a number of more than five employees, and collaboration with other local businesses. In the end, the 27 indicators have been grouped into the five predefined Booking.com impact categories (Fig 3). However, it should be mentioned that the practice “*Most food provided at the property is locally sourced”* has been re-categorised from “Energy and greenhouse gases” to “Destination and community,” for better representativeness.

Thus, sustainability practices verified through interviews and confirmed were assigned the value “1.” For the three selected indicators collected from the interviews, framed in the “Destination and community” category, their presence was indicated in the same way.

Subsequently, to gain a holistic perspective, all accommodation units were considered based on the aggregation formula 1 [33]. Each of the five categories was awarded a maximum of five points, regardless of the number of indicators present. The final values were then ranked in three main classes: high, medium, and low.


$$SPR=\sum_{i=\text{1}}^{k}\sum_{j=\text{1}}^{h_{i}}\frac{O_{m,i,j}}{h_{i}}\times F_{i}$$

### Aggregation formula 1 Sustainability practices ranking (SPR)

Where: SPR: final sustainability practices ranking of accommodation unit (m); m = 1,\...,n): index of the accommodation unit (with (n) the total number of establishments assessed); (k): number of sustainability categories, indexed by (i = 1,\...,k) (in our application: Waste, Water, Energy and greenhouse gases, Destination and community, Nature); (i): category index; (h_i): number of indicators within category (i); (j = 1,\l…,h_i): index of the indicators inside category (i).

Then, GIS techniques were used for the spatial representation of the accommodation units and of the sustainability practices ranking. In this regard, the postal address of each accommodation unit was obtained from Booking.com or their individual websites, then geolocated via Google Maps and represented using the ArcGIS Pro 2.5.0 software.

The fourth phase aimed to explore in depth the respondents’ perspective on sustainability beyond indicators and in connection with the territorial embeddedness of the accommodation unit in a protected area. Thus, several categories of opinions were identified by analysing the content of the narratives. In addition, two word clouds were elaborated to better emphasise the perceived benefits and downsides of developing a business within the SENP framework.

### Inclusivity in global research

Additional information regarding the ethical, cultural, and scientific considerations specific to inclusivity in global research is included in the Supporting Information (S1 Checklist).

## Results and discussion

Out of the total number of accommodation units registered on Booking.com in SENP, about 30% are declared as implementing sustainability practices. These outcomes are partially consistent with the results of other studies, which have found that the proportion of establishments on online booking platforms declaring eco-friendly measures in various destinations in several countries is significantly low [6,8,19,20]. Still, in comparison, SENP stands out with a significantly higher percentage, probably related to the natural protected area status and awareness.

The accommodation units reporting sustainability practices are territorially concentrated in a few spots (Fig 5), located mainly in the Covilhã, Manteigas and Guarda municipalities. Regarding the interviewed accommodations, there is a diversity of offers ranging from hostels and chalets to 4 and 5-star hotels. With the exception of four establishments, the rest opened after 2005, showing increased tourist offers in recent years. A noticeable trend is the opening of small family businesses 15–25 years ago, which, due to demand, have expanded over time. Also, some buildings with other original functionality have been refurbished into accommodation units. The lodging capacity varies from a minimum of 4–6 beds per unit to a maximum of 185 beds. Some establishments provide a variety of facilities (from restaurants to business centres, wellness, etc.), while others offer only basic services such as bed and breakfast. In addition, two have other integrated businesses, namely livestock farms, and another one stands out with an olive plantation, which tourists can visit. The number of employees ranges from one employee to a maximum of around 100 employees, of which 70 are permanent. However, the vast majority (80%) have up to 5 employees, some of which are the owners, underlining the family business character. The seasonality of tourism, with two peaks, one in winter when it snows and another in summer is highlighted as a shortcoming. However, some respondents mention a change in the wake of the pandemic, with an increase in the attractiveness of these areas in summer.

**Fig 4:**
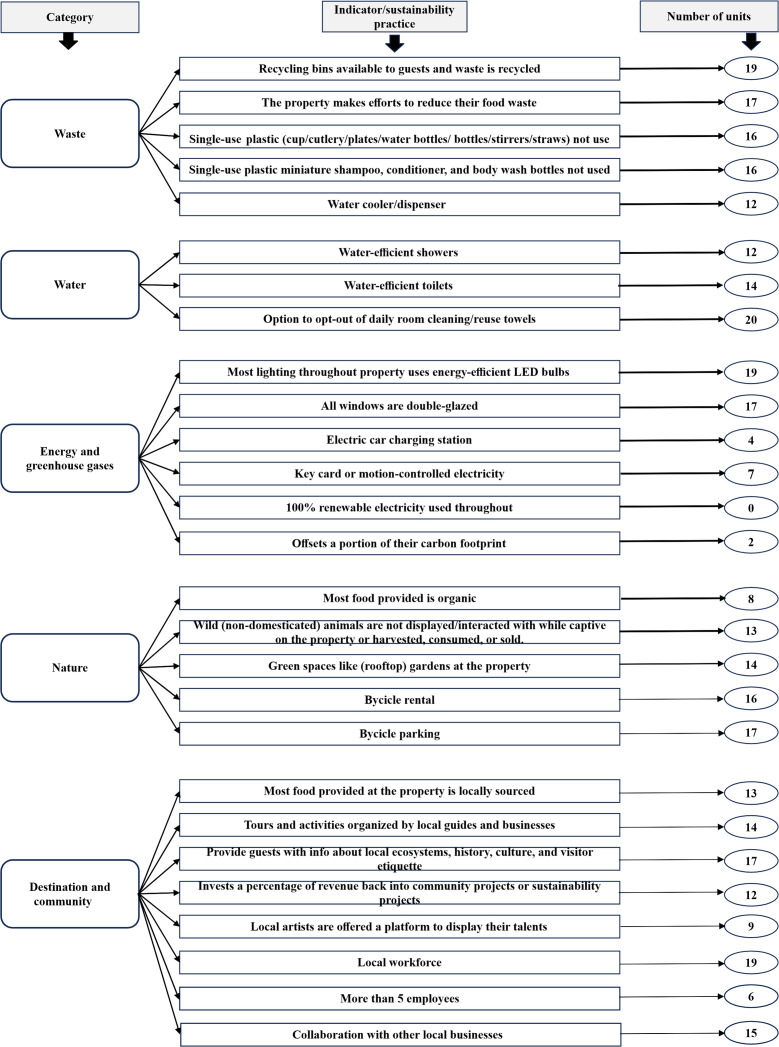
Degree of implementation of sustainability practices by the SENP accommodation units. Source: Booking.com and subsequent validation through face to face interviews.

The results of the sustainability practices declared by the accommodation units on Booking.com, confirmed and completed through the 20 interviews conducted are presented in Fig 4. The vast majority of the measures are confirmed by the interviews (90% of the total), attesting to the reliability of the reported information. Concerning unconfirmed practices, there are two situations: the first in which the interviews do not support the information presented on Booking.com and the second in which the indicator is not met, but there are some steps made towards what is stated. The first category includes the *water-efficient showers* and *water-efficient toilets* practices. From the second category, the *100% renewable electricity* measure was reportedly applied in five hotels. However, the interviews only partially confirmed this; they have photovoltaic panels, which provide only part of their consumption needs. A similar situation was found for two other practices: *the majority of the food provided is organic* and *locally sourced*, with some accommodations offering such products in small proportions.

At the same time, *recycling bins and waste is recycled* and *water-efficient toilets* practices are not reported on Booking.com but are highlighted in some interviews. In this regard, previous research advocates for better communication from the accommodation representatives [19,34].

Another result is that some practices do not have a clear frequency; for example, *Tours and activities organised by local guides and businesses* do not mean that they are offered permanently, but that they could be accessed under certain conditions. Thus, the information provided may be vague, offering only a general idea without specific details. Tourists must either be confident or seek more information on other online resources, if available, or by directly contacting accommodation, which is most often time-consuming. These results are consistent with previous studies, which highlight that, in general, on booking platforms, the information is scarce and must be checked directly on the hotels’ pages [20]. One of the reasons for this could be that in the wake of the pandemic, issues related to health and safety have been prioritised [6,20]. On the other hand, one of the benefits of using Booking.com is the ease of accessing data and the possibility of comparing the features of accommodation units in the same region to gauge progress.

Another noticeable aspect is that most of the variables focus mainly on environmental issues, a trend identified also in other studies addressing the relation between sustainability and the tourism sector [8,20].

Overall, the outcomes show that seven sustainability practices are the least implemented, of which four belong to the “Energy and greenhouse gases” dimension, one of which is not implemented anywhere, and two to the “Destination and community” dimension. At the opposite pole, with the best positioning, one measure related to Water is enforced in all the investigated units.

Our results are in some contrast with previous studies that have investigated the degree of implementation of sustainability practices in different regions through various methods. In a research focused on 12 accommodations in Catalonia, it emerged that most investments were made in the Energy field [15], while in Poland, most hotels implement measures related to Waste and Water, followed by Energy and greenhouse gases [6]. The results of the current study show that only two of the measures focused on energy practices are among the most implemented. In this regard, it partially supports the outcomes of Silik et al. [17], which found unsatisfactory results in terms of energy savings for accommodation units in protected areas in the Bolu province of Turkey. However, the same conclusion, which is not in line with our results, was also reached for water savings. Our findings align with the results of Rutecka et al. [6] regarding the frequent implementation of the measure *recycling bins and waste is recycled*, with Waste being the most represented category, followed by Water. This can be related to the fact that often the most common practices are those that are either easy to implement (e.g., recycling bins), or correlates with lower energy consumption (e.g., *energy efficient LED light bulbs*) [6,11] and therefore lower financial costs [15,18]. On the other hand, some practices are related to existing European Union regulations [13,15], such as reducing the impact of single-use plastic products.

These results confirm that variations can occur in the implementation of sustainability practices, rooted in place-specific factors [15] such as legally imposed rules, cultural values [13], business culture, environment policy [5], historical background, local network, etc.

**Fig 5:**
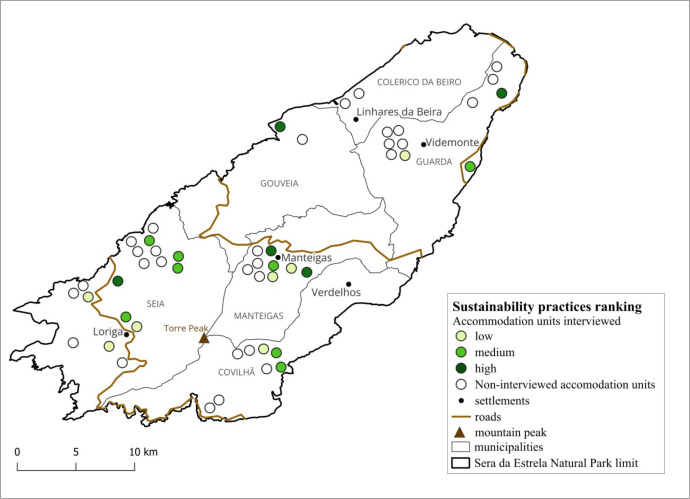
Sustainability practices ranking of the SENP accommodations units. Source: The boundary of Serra da Estrela Natural Park is based on European Protected Areas data from the European Environment Agency (https://www.eea.europa.eu/en/analysis/maps-and-charts/european-protected-areas-1) under CC-BY license. Interview locations and sustainability practices ranking represent original data created by the authors.

In terms of the sustainability practices ranking, 40% of SENP accommodation establishments that report this type of measure fall into the medium category, 35% into the low category, and only 25% into the high category. In the most problematic situation, one accommodation recorded the lowest score of only 3.9, far behind the next-ranked unit with a score of 10.8. Basically, the former implements very few measures, standing out with a passive attitude regarding sustainability: *“It’s important, but I don’t really do much”* (case 2). In other words, they have some green measures but are not too concerned about it. There is a diffuse territorial distribution of the units in the three categories, with no spatial clustering (Fig 5) (S1 Table).

The analysis of this sustainability practices ranking reveals that the accommodation units grouped in the high category display heterogeneous characteristics that cannot be directly associated with factors such as size, level of comfort, star rating, or age. Thus, this category includes both 4-star hotels and budget accommodations or guesthouses, a trend also found in the other two categories. This suggests that the implementation of sustainability practices depends more on operators’ business strategies, shaped by their values and beliefs, than on the structural characteristics of accommodation units.

In contrast, these findings are not consistent with other studies that show that larger and higher-class accommodations tend to implement more eco-friendly practices [13,14,35]. However, these studies have not focused specifically on protected areas, but on a larger geographical scale, at the national or regional level. Santos et al. [12], found from the literature review that geographical location influences the implementation of sustainability practices. In this regard, units with products and services reliant on natural heritage are more concerned with implementing these initiatives as part of a long-term commitment. Still, our results also contradict Font et al. [19], who analysed tourism enterprises in several natural protected areas in Europe and found that the larger and better star-rated ones have more actions implemented. A possible reason for these differences may be that this later study includes a wider range of tourism enterprises (e.g., restaurants) and is based on self-reported responses. Nevertheless, our results are in line with those obtained by Erdogan & Tosun [5], who analysing the environmental performance of accommodation establishments in a national park in Turkey, found that top hotels performed better on only seven out of 39 indicators considered. These contrasting results highlight the need for more empirical research focused on case studies from different places [16,17].

From the analysis of the sustainability practices ranking, one might assume that accommodations from the same category place similar importance on sustainability. Still, our results show that several differences can be highlighted based on the information collected through interviews, which provide more insight into the respondents’ approach and beliefs. Thus, seven of them perceive sustainability only in relation to their establishment, through an internal perspective based on the actions they can take, such as renewable energy projects or reducing consumption (e.g., water, energy).

The others stand out through a more holistic approach as they value sustainability both on an individual unit level and through the lens of the surroundings. This indicates an awareness that their business is territorially embedded into a specific natural environment that needs to be “preserved.” In this regard, one manager outlined that: *“Our main attraction is, of course, the park. So, if we destroy the nature around, we would be out of business. So, it would be of the most significant if we could preserve the nature”* (case 13). In the same vein, another respondent outline that *“the environment should be conserved...it’s good for business”* (case 19). These types of statements reflect a proactive attitude, interested in how they can contribute to achieving this goal of striking a balance between tourism development and conservation as a guarantee of the success of their business in the medium and long term. In the same line of thought, but in a more complex approach, another respondent argues that *“Our concern for sustainability is part of the business, even if the client isn’t interested in it. But, in reality, we realise that many of our clients come because of our policies. It’s part of our life ethic and, at the same time, it allows us, for example, to spend less in various areas of operation”* (case 3). This also reflects a business strategy based on personal values, which in the end brings benefits, highlighting that they can have both a prosperous and greener business. In this regard, several other studies identify as one of the drivers for adopting sustainable practices lifestyle values [4,16,36]. However, the primary motivation of tourism enterprises to engage in sustainable practices in several protected areas in Europe was found to be environmental protection [16].

On the other hand, regardless of the perspective, some also emphasise the importance of meeting client expectations and ensuring their satisfaction. In this respect, one manager stresses that concern for sustainability *“it’s very important...and it’s very necessary and also to attract tourists who really ask about this, like how sustainable are you?”* (case 18). This highlights an awareness of the requirements to demonstrate that they are eco-friendly, and in this respect previous studies have shown that sustainability practices can improve the establishment’s reputation [18] and can be a differentiating criterion for customers when the offer is similar [11,22,37]. At the same time, other research has also indicated that customer satisfaction is a major concern for entrepreneurs [4]. But this market pressure can go beyond the accommodation itself, also referring to the external environment, as another respondent stated that *“people are always concerned about the environment. And, nature is very important”* (case 4). In this regard, 80% of the respondents pointed out natural attractions as the main motivation for tourists to choose this area.

For two respondents, the commitment to being green also involves attempts to educate tourists by conveying a concern for the responsible use of resources and reducing consumption. Meanwhile, others focus on environmental education, as *“we really want to get people back into nature and appreciate nature more”* (case 18). Therefore, there is a concern about tourists’ behaviour and increasing their awareness of different aspects of sustainability. The results are consistent with other studies that emphasise the role of hosts in informing guests about their responsibilities in caring for the environment [10,15], which could eventually lead to lower consumption and reduced costs [19].

Overall, the analysis of respondents’ perceptions indicates that commitment to sustainability is driven by a primary reason (e.g., environmental protection, lifestyle values), often intertwined with secondary motivations. However, the results are consistent with the assertion made by Armas-Cruz et al. [18] that the adoption of these practices primarily stems from the voluntary commitment of the owners/managers.

It is found that other sustainability practices are applied that are not listed in Booking.com, such as the impact on the local economy through collaboration with other businesses, educational activities, employment for the community, biomass production for heating, rainwater harvesting, spring water capture, water recycling, using food waste as fertiliser, etc. Also, mobility and, more specifically, access to public transport remains an unaddressed issue on Booking.com, whether we are talking about tourists or employees.

These results confirm the concerns expressed in previous research that not all initiatives can be communicated on the platform [20]. On the other hand, they highlight a limitation of Booking.com that does not offer the possibility to integrate other sustainability practices, especially from an economic and social perspective.

A significant finding from the interviews was that the overwhelming majority of respondents relate to sustainability only through the lens of the environmental pillar. This highlights a pro-environmental attitude, whether it relates to sustainability practices and their impact on their accommodation units or in relation to preserving the surrounding natural features. These results may be due to the fact that they are more aware of the environmental features because they operate in an NP area and also in relation to attracting nature-loving tourists or because there are clear economic implications that can lead to cost savings. For some respondents, a possible link may also exist with the growing awareness of the environment’s importance following the degradation of certain areas by forest fires. The most recent fire, which occurred in 2022, affected a considerable area in the central-eastern part. Moreover, seven of the respondents perceive the fires as a major risk for tourism activity given that *“before we had some big fires in the park when we had the best nature, we had a lot of foreign tourists”* (case 13).

This environmental-centric view may lead to an incomplete approach to sustainability, as true sustainable development requires a balance between environmental integrity, social needs, and economic viability [38]. Such perspective can result in an incomplete understanding and potentially conflicting interpretations of sustainable development [39], overshadowing local identity and cultural dynamics [40]

**Fig 6:**
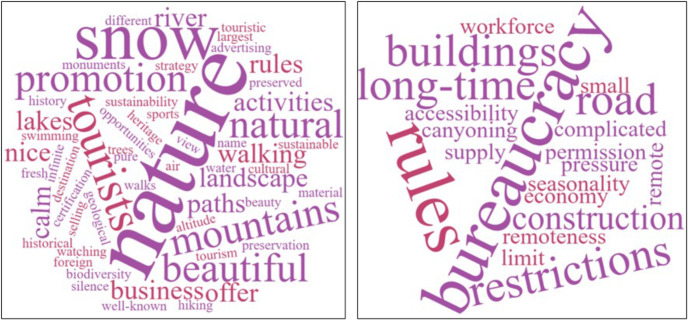
Word clouds with benefits (a) and downsides (b) perceived by accommodation representatives for the development of their business in the SENP.

Two word clouds (Fig 6) reveal the main issues indicated in the entrepreneurs’ discourses related to developing their business within the SENP in terms of perceived benefits and downsides for practising sustainable tourism. Regarding the potential benefits of operating their business in the SENP, responses can be divided into three major categories. The first group of views focuses mainly on natural capital, indirectly linked to protected area status granted precisely as recognition of these predefined attributes. Thus, they associate the SENP with “nature” as a whole or with certain natural assets, such as “rivers,” “lakes,” or an unpolluted area defined by “fresh air” or “pure water.” Building on these resources, some highlight the possibility of outdoor recreational “activities” such as “walking,” “swimming,” etc. From a more business-oriented perspective, a manager sees nature in its complexity and diversity, from which economic opportunities are manifold. In this regard, he stated that: *“The offer is good, the quality is good because we are selling nature...And we have thousands of different business opportunities for different audiences because nature is infinite”* (case 6). For another respondent this mountain area offers a comparative advantage for niche tourism services as an alternative to coastal areas in Portugal, considering that *“it is very important to offer something different from having a beach”* (case 5). More specifically, some have found the potential to carve out a niche given that it is one of the few places in Portugal where it snows, so *“every time that snows, we have lots of clients”* (case 17).

The second category of opinions appreciates the NP label as a well-defined brand identity, which in itself brings “promotion,” attracts visitors, and, in this way, supports business development. In this regard, one respondent mentioned that *“this area is well-known in Portugal  ...and that can bring a lot, a lot of people...If we were in another place and not a big natural park, for sure we would not have so many visitors”* (case 13). The geographical location is considered *“very important because the natural park itself is a destination”* (case 12). From a deeper perspective, through a place-based approach, another respondent stresses that he designed his business by leveraging endogenous resources: *“Our business strategy is largely defined precisely because we are in a natural park. Many of the activities are built around and aimed at publicising the park’s natural and cultural heritage”* (case 3).

These two major categories of views are often intertwined, highlighting SENP as a catalyst for business development. It is also noticeable that only one respondent refers to SENP as an institution, identifying the rules that protect nature.

On a discordant note, two respondents expressed an indifferent opinion as they see no benefit in developing their business in the SENP.

In terms of the downsides of business development in the SENP, there are two contrasting views. The first group of respondents reveals a number of impediments (Fig 6), of which the most frequently mentioned are the restrictions/rules imposed by the NP administration, which they have to comply with, especially regarding the construction of new buildings or the restoration of old ones in relation to the original building (case 2). One person also indicates limiting certain activities (e.g., canyoning) carried out for tourists to certain periods for the conservation of certain habitats and species (case 8). Another issue is bureaucracy, which means a long time to obtain the necessary permissions from an additional administrative body. Other perceived disadvantages are somehow indirectly linked to the SENP, i.e., location in a remote area and related accessibility problems, as well as an economy based largely on seasonal tourism.

Taking a different approach, the second group, formed by ten entrepreneurs, sees no downside in developing their business in SENP. For some of them, although they mention the existence of rules, these are considered “normal,” or even labelled as “good restrictions” and “necessary” to protect the environment and preserve natural beauty and biodiversity. Moreover, considering an integrative perspective of nature activities and tourist attitudes, two entrepreneurs stress the need to be cautious as this is a “sensitive place.” In this regard, one of them points out the need for conscious tourists who *“have to understand where they are and take advice, don’t leave rubbish and so on. For instance, in winter, when it snows, there is a lot of pressure from cars, people....in the same place”* (case 14). The other respondent, both in terms of business development and tourists, emphasised the need for an entity to impose rules because *“there are things that need to be managed and controlled”* (case 4).

For other respondents, from a sectoral perspective, the strict rules are seen as a means to safeguard the businesses by preventing potentially chaotic development of the built-up area because: *“we’re not afraid of big buildings being built right next to us”* (case 12). In the same line of thoughts, another respondent emphasises: *“As far as building rules go, it’s pretty hard. But it’s also normal because they have to make sure that when you’re inside the natural park, you don’t build anything, anywhere. Also, that when you have permission to build you respect the style of building in the area, with natural materials”* (case 16). These results are consistent with the challenges identified in the SENP management plan as requiring special attention: tourism pressure, uncontrolled urbanization and threats to biodiversity [26].

Regarding interactions with SENP representatives, eight respondents do not keep in touch with them, eight contact them only occasionally and only four collaborate more often. Of those interviewed, six perceive the NP institution only as a regulatory body that they have to inform about certain activities and ask its permission. Still, an active partnership is indicated by three establishments, to whom they provided “books/studies” (case 3), organised trips for tourists or advertisements. However, most seem to have a passive relationship with SENP representatives, where they do not make contact with them or rarely collaborate. On the whole, there are several criticisms, with four respondents indicating that they do not perceive their presence in the territory. Some reasons for this situation extracted from the opinions of other respondents are the inertia of public institutions, lack of funds and staff, and even lack of power, as some issues are the responsibility of municipalities. The expectations for the SENP are related to creating preconditions for tourism development in the area, such as environmental education for the staff, forest management, maintenance of hiking trails, etc.

These findings align with other studies that highlight tourism stakeholders’ dissatisfaction with their relationships with the management of protected natural areas, rooted in a top-down approach [7,41,42]. However, these outcomes mirror previous research denoting differing views and interests of various stakeholders concerning nature-based tourism and sustainability within the same territory [43–48].

This paper highlights a new perspective emphasising the role of accommodation units as promoters and beneficiaries of sustainable tourism within natural protected areas. Furthermore, the research raises awareness of a rarely discussed topic: whether entrepreneurs’ commitment to sustainability is influenced by the local context, more precisely by their location in a natural protected area, subject to specific regulations. Integrative analysis of the interviewees’ responses shows that most of them have a broader, multi-scalar perspective on sustainability through the lens of embeddedness in this specific type of territory. This reinforces the findings of other studies, which conclude that the reasons for pro-sustainability attitudes are diverse [16] and also depend on geographical context [12,18]. Deepening the perspective, some entrepreneurs seem to have a more practical motivation related to the valorisation of valuable natural capital that must be preserved while others are guided by personal values and beliefs. It has been found that entrepreneurs perceive nature as a resource [46] whose outstanding value is validated by its status as a protected area, further promoted as a tourist destination with a well-known identity. This status gives it a competitive territorial advantage [18] over surrounding areas. In the same vein, some authors talk about sustainability as a business opportunity, through the promotion of sustainable services [48] and profit generation [7]. A recent study aimed at identifying prospective trends in nature-based tourism, based on expert opinions from various areas in Europe and the western United States, outlines five key categories. One of these categories focuses on sustainability while another emphasises health, more specifically, outdoor activities that can be carried out in nature [41]. Additionally, the study highlights digitalisation as a crucial factor for promotion and business efficiency, especially in remote places, a point also noted by other researchers [45].

Overall, these findings are in line with other studies emphasising that NP status may represent a significant instrument for regional development [49,50], bringing economic vitality [48,51]. Entrepreneurs are increasingly interested in contributing to the preservation of natural heritage for long-term benefits. However, some feel pressured by the growing number of tourists and the interest from potential investors in future projects. They are concerned that without clear measures in place, degradation of natural resources may occur. In this context, they acknowledge the significance of NP administration as a guarantor of preserving natural resources, which is a shared concern. These results contrast with other studies revealing that stakeholders tend to have positive opinions about nature preservation and the benefits of NP status in general, particularly if they are involved in its management and governance [7,44]. Our outcomes indicate that while most entrepreneurs lack an active partnership with NP representatives, they remain focused on sustainability. Perhaps this is because it is an isolated area where tourism is one of the few viable economic alternatives. As proved in other studies, in these destinations, reliance on tourism grows as traditional activities decrease [52].

The predominantly environmental perspective on sustainability embraced by accommodation representatives, along with the NP administration’s primary focus on environment-centric actions and regulations, can create a solid foundation for comprehensive sustainability development within SENP. This common ground can serve as a crucial starting point for a balanced use of resources [45] in the medium and long term, in a region with growing tourist appeal. Furthermore, it can foster local development by enhancing the socio-economic perspectives of local communities [53] and by valuing and preserving traditional heritage, activities, and customs.

In this context, NP administration should recognise the importance of understanding entrepreneurs’ perceptions to establish a viable partnership in the medium and long term [44]. Previous studies have emphasised cooperation as a powerful tool for achieving sustainability with mutual benefits for all parties involved [18,23,43] and for mitigating disagreements [54,55]. Furthermore, synergies between actors are advocated to enhance outcomes and benefit-sharing from tourism activities [56]. In light of this, we advocate for systemic transformation through a shared vision and an integrative approach of different stakeholders [45] to balance nature preservation with sustainable tourism development. Establishing an active partnership rooted in dialogue, understanding diverse viewpoints, and fostering trust is seen as essential for effective cooperation [41]. This involves sharing knowledge, including providing education on sustainability to accommodation representatives, enabling them to promote it to tourists, generating spillover effects with medium- and long-term positive impact. In the same vein, Coll-Barneto & Fusté-Forné (2023) highlighted the importance of accommodation sector actors in promoting sustainable behaviour among tourists [15]. This is especially important in the post-pandemic context, where tourism is considered an opportunity [51] to enhance livelihoods in many protected areas, particularly in remote regions.

This study provides valuable insights, but it has some limitations. Firstly, the interviews were conducted with only 20 representatives of accommodation units due to the seasonality of the tourist offer and participant availability, which may limit the range of viewpoints presented. Then, the research focuses on a particular group of stakeholders from one natural protected area, which may limit the generalizability of the findings. These limitations emphasise the need for further empirical research in various contexts. This would contribute to a better understanding of stakeholders’ commitment to sustainability practices and to enhancing the existing knowledge on sustainable tourism in natural protected areas, providing a comprehensive approach.

## Conclusions

This study investigated the engagement of accommodation sector representatives from Serra da Estrela Natural Park in sustainability practices through a multi-stage research approach. As a novel contribution, the measures reported by accommodation units on the popular Booking.com platform were subsequently verified through interviews with their representatives. By addressing a recognised knowledge gap, this research validates the online information provided on this platform, revealing its usefulness as a reliable tool for gaining insights into the sustainability practices implemented in a given territory.

Studies investigating green practices in different places are generally characterised by heterogeneity of indicators that do not allow a comparable analysis. At the same time, it often involves considerable resources. As an alternative, using data from this platform can be a cost-effective way to simultaneously analyse larger regions or to examine the dynamics of the same area. Still, there needs to be a broadening of the platform’s general framework to include other sustainability practices that were found in the interviews to be in place but not reportable. Additionally, a drawback is that Booking.com only lists practices without offering further information, necessitating additional research by tourists.

Sustainability practices ranking reveals that 25% of the accommodation units in SENP fall into the high category, which is a rather low percentage, especially considering their location in a protected area (S1 Table). An important finding is that even if it seems that accommodation establishments in the same category have similar approaches based on their ranking, in fact the interviews reveal significant differences. In this regard, most respondents also perceive sustainability in terms of attributes of the external environment, which is understood almost entirely as a concern for preserving natural features of the SENP. In their case, a strong link was observed between the success of their business and the preservation of nature, given that tourists choose this area mainly for its natural attractions. This reveals a systemic approach to sustainability, with the accommodation unit perceived as an element embedded in the wider NP framework. In some cases, this attitude also reflects personal values and beliefs.

Thus, by deepening our analysis, the current research provides significant insights into the commitment of accommodation establishments’ representatives to sustainability in protected areas, contributing empirical evidence to bridging this recognised research gap. We found that the overwhelming majority of respondents perceive sustainability through the lens of the environmental component and not in terms of socio-economic considerations. This denotes overall a pro-environmental attitude, even though the degree of implementation of sustainable practices reflects commitment of varying extents.

In Portugal, tourism in protected areas is defined as a strategic priority. These specific territories are becoming more attractive, with significant anthropic pressure in some areas, including in SENP, especially in winter. In this context, we are advocating for the enforcement of clear policies to encourage the practice of sustainable tourism and also for urgent measures to gain more support from the NP institution. The findings highlight that most respondents perceive several benefits of locating their business in a protected area and are open to collaboration with its administration as a foundation for strategic tourism planning. However, currently, the relationship with the SENP representatives is largely passive, characterised by occasional or no interaction.

Overall, the ambivalent character of the respondents’ perception of SENP is noted: they understand it either as a territory with outstanding natural setting in which their business is embedded or as a label, per se, behind which there is an institution imposing rules/restrictions. Still, a notable outcome is that half of the respondents perceive regulations not as downsides but as necessary ways to prevent chaotic development and environmental degradation. Additionally, our findings do not align with previous research indicating limited development opportunities due to imposed NP restrictions. However, only 30% of accommodation units report sustainability practices, so it is important to understand the perspective of other tourism stakeholders. To this end, we advocate for the elaboration of integrated development measures involving all key actors.

The interview-based findings yield a deeper perspective on sustainability than the characteristics synthetically mirrored on Booking.com, which denotes the significance of gaining more insights at the local level. Furthermore, the study contributes to raising awareness of the complexity of the sustainable tourism approach, particularly in protected areas. Incidentally, given that the results are only partly consistent with previous research it demonstrates that the local context matters and the approach can be used for future research in other protected areas. In this regard, the outcomes can be a starting point for further comparative analysis.

Future studies may aim at understanding the perspective of the representatives of accommodation establishments that do not declare green practices on Booking.com. Further research can approach accommodation units in other protected areas to investigate whether the accuracy of the information reported on the online platform is confirmed in similar contexts and even beyond to reveal if the results can be generalised.

## Supporting information

S1 Checklist Inclusivity in global research questionnaire. (DOCX)

S1 Table Sustainability practices ranking. (DOCX)
