# Supplementary material for: Sustainability practices of accommodation sector representatives: The case of mountain protected area Serra da Estrela Natural Park (Portugal)
Source: PLoS One. 2026 Apr 20;21(4):e0347472. doi: 10.1371/journal.pone.0347472 (PMC13094967; doi:10.1371/journal.pone.0347472)
Supplement: S1 Table — (DOCX) [file pone.0347472.s002.docx]

| **Sustainability practices ranking (limits)** | **Number**  **of accommodation units** | **% of accommodation units** |
| --- | --- | --- |
| High (>20) | 5 | 25 |
| Medium (15 to 20) | 8 | 40 |
| Low (<15) | 7 | 35 |

**Sustainability practices ranking among interviewed accommodations units**
